# Cytokine-Induced Memory-Like NK Cells: From the Basics to Clinical Applications

**DOI:** 10.3389/fimmu.2022.884648

**Published:** 2022-05-04

**Authors:** Iñigo Terrén, Ane Orrantia, Gabirel Astarloa-Pando, Ainhoa Amarilla-Irusta, Olatz Zenarruzabeitia, Francisco Borrego

**Affiliations:** ^1^ Immunopathology Group, Biocruces Bizkaia Health Research Institute, Barakaldo, Spain; ^2^ Ikerbasque, Basque Foundation for Science, Bilbao, Spain

**Keywords:** NK cells, memory-like, trained immunity, cancer immunotherapy, AML - acute myeloid leukemia, cytokine-induced memory-like NK cells, adaptive NK cells, adoptive cell therapy (ACT)

## Abstract

Natural killer (NK) cells are lymphocytes with a key role in the defense against viral infections and tumor cells. Although NK cells are classified as innate lymphoid cells (ILCs), under certain circumstances they exhibit adaptive and memory-like features. The latter may be achieved, among others, by a brief stimulation with interleukin (IL)-12, IL-15 and IL-18. These cytokine-induced memory-like (CIML) NK cells resemble the trained immunity observed in myeloid cells. CIML NK cells undergo transcriptional, epigenetic and metabolic reprogramming that, along with changes in the expression of cell surface receptors and components of cytotoxic granules, are responsible for their enhanced effector functions after a resting period. In addition, these memory-like NK cells persist for a long time, which make them a good candidate for cancer immunotherapy. Currently, several clinical trials are testing CIML NK cells infusions to treat tumors, mostly hematological malignancies. In relapse/refractory acute myeloid leukemia (AML), the adoptive transfer of CIML NK cells is safe and complete clinical remissions have been observed. In our review, we sought to summarize the current knowledge about the generation and molecular basis of NK cell memory-like responses and the up-to-date results from clinical trials with CIML NK cells.

## Introduction

Natural Killer (NK) cells are cytotoxic and cytokine producing lymphocytes classified within the innate lymphoid cells (ILCs) family (1–3). They can be a useful tool in cancer immunotherapy due to their antitumor functions that do not require prior sensitization. Indeed, a relevant number of therapeutic strategies have been developed to exploit NK cell potential and some of them are currently being tested in multiple clinical trials (4–9). These therapeutic approaches are focused on improving cancer patients’ NK cell effector functions by inducing their activation with cytokines or with bi/tri/tetra-specific killer cell engagers and/or on preventing NK cell inhibition with immune-checkpoint inhibitors, such as monalizumab that blocks the inhibitory receptor CD94/NKG2A (hereafter NKG2A) (6, 10, 11). In addition, the adoptive transfer of cytokine-preactivated or genetically-modified NK cells have been found to be relatively safe and have shown great potential in cancer therapy (9, 12–15).

Historically, NK cells have fitted in the definition of innate immunity: short-lived cells capable of mounting a rapid antigen-independent response (16). However, a marked paradigm shift has occurred in the field since the discovery of a long-lived subpopulation of NK cells able to mediate hapten-specific recall responses (17). Although not completely well understood, the existence of adaptive and memory-like NK cells were subsequently reported in response to other haptens and viruses in different species, including mice, non-human primates and humans (18). Interestingly, it has also been reported that stimulating NK cells with interleukin (IL)-12, IL-15 and IL-18 can endow them with memory-like properties (19). Here, we summarize the current knowledge about memory-like NK cells, focusing on IL-12/15/18-induced memory-like NK cells, and discuss their properties and therapeutic potential.

## The Diversity of Adaptive, Memory and Memory-Like NK Cells

The term “natural killer” encompass a diverse and versatile group of cell subsets that show differences in their phenotype and effector functions (20). These subsets, though, are not static. On the contrary, they can be modulated under certain circumstances that led to cell activation and, furthermore, they can acquire memory-like properties after a first activation event. These findings have encouraged a reevaluation of the definition of the “immune memory”, which will probably require to be updated as new discoveries are made. Understanding memory-like features of innate immune cells can be challenging due to the diverse contexts and experimental settings in which they have been studied. Some memory/adaptive NK cells have been described to show an increased activation upon restimulation with the same stimuli they were previously exposed. In contrast, other memory-like NK cells exhibited an enhanced activation following restimulation with a variety of stimuli, thus having a non-specific response (8). Interestingly, some of the NK cell memory-like responses that have been studied until now fit into the definition of trained immunity usually referred to a memory program found in other innate immune cells. According to a recent definition, “trained immunity” would describe a behavior in which a first stimulus increased the functional status of the innate immune cells, that later return to the basal activation state and have the ability of reaching higher functional status upon a second challenge (21). Thus, it could be worthwhile to explore the similarities between the memory programs of different innate immune cells.

**Figure 1:**
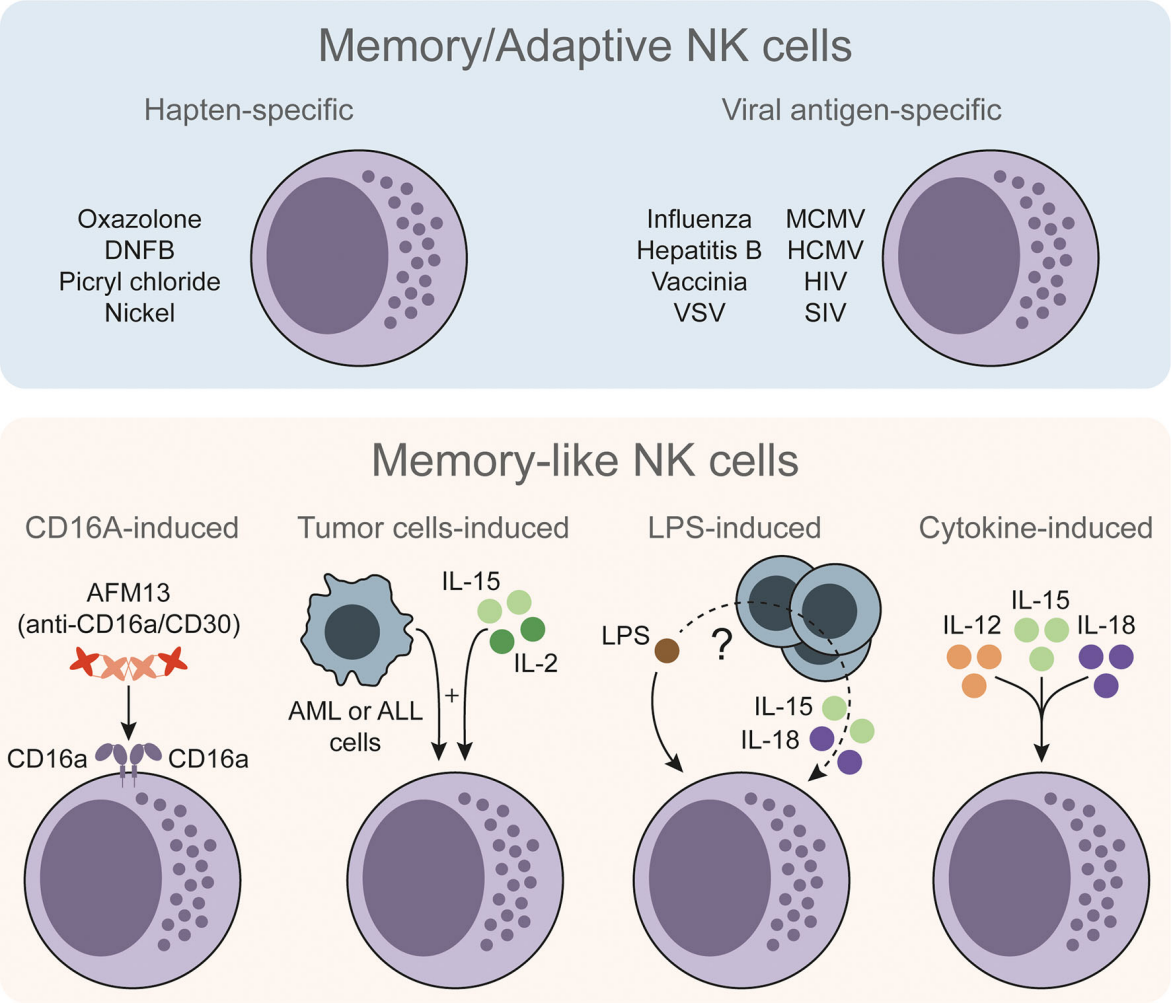
The diversity of memory/adaptive and memory-like NK cells. Memory/adaptive NK cells with antigen-specific responses have been described in different models such as contact hypersensitivity, viral infections or vaccinations. Memory-like NK cells show an increased but less specific response upon restimulation. These memory-like NK cells could be trained through CD16a-induced stimulation or by stimulating NK cells *ex vivo* with certain tumor cells and low doses of IL-15 and IL-2. In an *in vivo* setting of LPS-induced endotoxemia, LPS can endow NK cells with memory-like properties through non-well defined mechanism. NK cells can be stimulated directly with LPS plus IL-15 and IL-18, and other cells (e.g. DCs) may contribute to the NK cell training process. NK cells can be also stimulated with IL-12, IL-15 and IL-18 to endow them with memory-like properties. NK, natural killer; DNFB, 2,4-dinitrofluorobenzene; VSV, vesicular stomatitis virus; MCMV, murine cytomegalovirus; HCMV, human cytomegalovirus; HIV, human immunodeficiency virus; SIV, simian immunodeficiency virus; AML, acute myeloid leukemia; ALL, acute lymphoblastic leukemia; IL, interleukin; LPS, lipopolysaccharide.

NK cells with memory/adaptive and memory-like features have been reported in response to a wide variety of stimuli (**Figure 1**). First evidences were described in response to haptens (17, 22). Of note, type 1 ILCs (ILC1s) have also been reported to acquire hapten-specific memory potential (23), so the actual contribution of NK cells versus ILC1s during these memory responses is still unclear. NK cells with antigen-specific responses have been better characterized during certain viral infections (18, 24, 25). Moreover, since NK cells can be activated by different means, memory-like properties have been also described in other antigen-independent approaches. For instance, CD16a-induced activation can prime NK cells and enhance their interferon gamma (IFNγ) production upon restimulation (26). In a different setting, Rasid et al. showed that mouse NK cells are activated in an endotoxemia model and return to a resting state after 14 days. Furthermore, these pre-activated NK cells showed an increased IFNγ production upon lipopolysaccharide (LPS) restimulation (27, 28). It is interesting to note that previous works have shown the ability of dendritic cells (DCs) to prime NK cells (29), and highlighted the role of DCs in activating NK cells during LPS-induced inflammatory conditions (30). Therefore, interacting with other cell types may endow NK cells with memory-like features. Indeed, tumor-primed NK cells showed an increased anti-leukemic activity when re-exposed to cancer cells (31). Other authors also have studied the priming effect of certain tumor cell lines (32–34) and feeder cells (35) on NK cells, but failed to describe a recall response after a resting period. Finally, cytokine-induced activation has been extensively described to have a priming effect and, under specific experimental conditions, to endow NK cells with a memory-like phenotype that resembles trained immunity.

## Cytokine-Induced Memory-Like (CIML) NK Cells

Numerous studies have addressed the activating effect that cytokines have in NK cells, and more importantly, the synergistic effect that certain cytokine combinations achieve. A particularly relevant case is the combination of IL-12, IL-15 and IL-18, which can induce memory-like properties in NK cells. First evidences of this effect were reported by Cooper et al. in 2009. They found that rested murine NK cells that were previously stimulated for a short time with IL-12/15/18 exhibited higher IFNγ production following cytokine restimulation, compared to non-preactivated NK cells (19). Three years later, Romee et al. described similar enhanced responses in human NK cells (36). Since then, many authors have extensively studied these IL-12/15/18-preactivated or cytokine-induced memory-like (CIML) NK cells in an effort to understand their biology and how can they be exploited for cancer immunotherapy.

### Distinctive Features of CIML NK Cells

The ability of mounting an enhanced response upon a second stimulation makes CIML NK cells fascinating, but not unique. As mentioned before, memory-like behavior of NK cells has been described in different settings. So, what else makes CIML NK cells different from conventional NK cells? Initial studies on IL-12/15/18-preactivated NK cells described that, besides an enhanced reactivity, there are many changes in their phenotype (37). Some of them, as it is the case of semaphorin 7A (SEMA7A), may be linked to the enhanced responses of CIML NK cells. The authors suggested a novel mechanism through which CIML NK cells could form conjugates mediated by the interaction of SEMA7A and its functional ligand integrin-β1 that could help maintaining their increased functionality (38). An interesting observation was that CD16 expression was reduced following stimulation with cytokines, a process described to be mediated by the metalloproteinase ADAM17 (39). Strikingly, although CD16 expression is reduced in CIML NK cells, they preserve the capacity to mediate antibody-dependent cell-mediated cytotoxicity (ADCC) (40–43). Another remarkable characteristic of CIML NK cells is the increased expression of the α chain of the high-affinity IL-2 receptor, also known as CD25, which has been reported to be upregulated following stimulation with cytokines (41, 42, 44–49). Due to this feature, CIML NK cells are able to proliferate in the presence of low doses of IL-2 (44, 45). It is interesting to note that, while CIML NK cells retain a relatively high expression of CD25 for a certain period of time, they partially recover the downregulated CD16 expression during the following days after the cytokine preactivation (42, 50). These findings imply that some of the phenotypic changes described in CIML NK cells could have a transient nature. Indeed, this is the case of KIR2DL2/L3, KIR2DL1 and KIR3DL1 receptors, whose surface expression has been described to be downregulated following IL-12/15/18 stimulation and restored after 3 days in the presence of IL-2 (41). Hence, considering the memory-like behavior of IL-12/15/18-preactivated NK cells, it is interesting to study what features change over time and what characteristics could be permanent.

Following stimulation with cytokines, NK cells modify their metabolic activity as a consequence of their transition from a resting to an activation status (51). Specifically, IL-12/15/18-stimulated NK cells upregulate the expression of certain nutrient transporters, including the transferrin receptor CD71, the heavy subunit of multiple heterodimeric amino acid transporters CD98, and glucose transporters GLUT1 and GLUT3, and retain an elevated expression of CD98 and GLUT1 after a resting period (52). Moreover, upon IL-12/15/18 stimulation NK cells increase their metabolic activity and retain a metabolic profile shifted towards glycolysis for at least 7 days (52–54). Interestingly, inhibiting glycolytic activity with 2-DG during IL-12/15/18 stimulation resulted in a lower IFNγ production, both immediately after stimulation with cytokines and after a resting period of 6 days (53, 55). Similarly, it has been shown that inhibiting oxidative phosphorylation and preventing the activation of the Srebp transcription factor during IL-12/15/18 stimulation also limited the enhanced functionality of CIML NK cells (55, 56). These findings suggest that NK cells have specific metabolic requirements during their “training” or preactivation phase that are crucial for the acquisition of their memory-like properties.

Trained immunity has been described to rely on functional modifications, metabolic and transcriptional reprogramming and epigenetic remodeling (57). These epigenetic changes are heritable and can be based on either DNA methylation or histone modifications, including acetylation and methylation. These modifications have an impact in chromatin structure and thus can regulate the accessibility to transcription factors. Unfortunately, the epigenetic modifications induced by IL-12/15/18 stimulation in NK cells have been poorly characterized. It has been described that there is an epigenetic remodeling in different adaptive and memory-like NK cell models, such as those induced by viral infections, contact hypersensitivity, or LPS-induced endotoxemia (58). On the other hand, although the combination of IL-12/15/18 stimulation was not tested, Wiedermann et al. demonstrated that cytokine-induced signaling *via* STAT proteins showed distinct modes of epigenetic regulation (59). Thus, it is tempting to speculate that CIML NK cells could follow similar mechanisms to retain their memory-like properties. One of the few evidences supporting this hypothesis was reported by Ni et al., who found that the conserved non-coding sequence 1 (CNS1) in the *Ifng* locus of IL-12/15/18-preactivated NK cells was demethylated 11 days after transferring these cells into RAG^-/-^γc^-/-^ mice (60). Demethylated CNS1 in the *Ifng* locus was also found in human CIML NK cells after a resting period of 14 days (54). Additionally, it has been reported that the histone methyltransferase EZH2 plays a crucial role in CIML NK cells, since its pharmacological inhibition with UNC1999 limited the enhanced IFNγ production following IL-12/18-restimulation (61). These reports suggest that there are some epigenetic changes accompanying functional, transcriptional and metabolic reprogramming of IL-12/15/18-stimulated NK cells. Whether CIML NK cells have a particular epigenetic imprinting, and its relevance is still to be determined. Future studies will fill this gap in the knowledge.

### CIML NK Cells in the Pre-Clinical and Clinical Stages

Due to the increasing interest of NK cells in the field of cancer immunotherapy and having described an enhanced IFNγ production upon cytokine restimulation of CIML NK cells, the next logical step was to evaluate their potential to kill tumor cells. Early studies reported that control and CIML NK cells exhibited similar ability to kill YAC-1 lymphoma targets after a resting period *in vitro* (19), and also showed no difference in degranulation when co-cultured with these target cells after a resting period *in vivo* (62). Interestingly, restimulation with YAC-1, 721.221 or K562 targets cells resulted in an enhanced IFNγ production of CIML NK cells (36, 45, 50, 52, 60, 62–64). Increased IFNγ production, but not enhanced cytotoxicity, were also reported when CIML NK cells were restimulated *in vitro* with a variety of hepatocellular carcinoma cell lines after a resting period (65). Similar results were obtained when CIML NK cells were restimulated *in vitro* with ovarian cancer cell lines, showing identical degranulation activity and increased IFNγ production, but an enhanced specific killing of SKOV3 ovarian tumor cells (50). Hence, *in vitro* studies confirmed that the previously described cytokine restimulation-induced IFNγ production could be also found when CIML NK cells are restimulated with different target cells. Nonetheless, it was necessary to explore the effect of IL-12/15/18-preactivated NK cells *in vivo* to better understand their potential therapeutic effect and, excitingly, these studies revealed promising results.

Ni et al. found that the adoptive transfer of IL-12/15/18-preactivated NK cells in combination with radiotherapy resulted in a reduced growth of established tumors and increased survival in a murine model. Authors demonstrated that increased IFNγ and perforin levels of CIML NK cells were crucial for their therapeutic effects (46). Of note, IL-15- and IL-12/15/18-preactivated NK cells did not show any antitumor activity without radiotherapy, which has been described to support NK cell engraftment (46). Similarly, it was later reported that the adoptive transfer of CIML NK cells in combination with radiotherapy slowed down tumor development in a rat model of T cell acute lymphoblastic leukemia (48). These reports evidenced the therapeutic effect of CIML NK cells under the appropriate conditions and highlight the necessity of studying their antitumor properties under different experimental settings. In line with this idea, it has been described that the adoptive transfer of CIML NK cells to lymphoma-bearing mice did not improve their survival. However, tumor progression was significantly delayed when CIML NK cells and A20 lymphoma targets were simultaneously co-injected (47). Other authors have also confirmed that survival of A20 cell-bearing mice was similarly improved by the infusion of IL-12/15/18-preactivated NK cells (49). CIML NK cells also showed an effective control of tumor growth in a mouse model of multiple myeloma (66). Strikingly, therapeutic effects of IL-12/15/18-preactivated NK cells were not only reported in hematologic malignancies. Sub-lethal irradiation followed by the adoptive transfer of CIML NK cells significantly decreased tumor growth in a mouse model of ovarian cancer (50). IL-12/15/18-preactivated NK cells were also reported to be able to localize in the liver and exert their antitumor activity in a mouse model of hepatocellular carcinoma (65). Moreover, Ni et al. reported that IL-12/15/18-preactivated NK cells exhibited therapeutic effects analyzing lung metastases in a melanoma mouse model (46). More recently, Marin et al. described that CIML NK cells were able to control tumor burden in another mouse model of melanoma (67). Collectively, these reports strongly suggest that the adoptive transfer of CIML NK cells could have considerable therapeutic benefits in certain cancer patients.

Having established the rationale to explore the antitumor effect of IL-12/15/18-preactivated NK cells in the clinic, different clinical trials were initiated. Currently, there are a total of eight ongoing clinical trials in which the safety and therapeutic effects of CIML NK cells are being tested in patients with different malignancies, including acute myeloid leukemia (AML), myelodysplastic syndromes, multiple myeloma, and head and neck squamous cell carcinoma (NCT01898793, NCT02782546, NCT03068819, NCT04024761, NCT04290546, NCT04354025, NCT04634435, and NCT04893915, from clinicaltrials.gov). In 2016, it was reported that CIML NK cells were able to control K562 tumor growth in a leukemia mouse model, which was translated in a prolonged survival. Moreover, authors described that IL-12/15/18-preactivated NK cells showed graft-versus-leukemia effect in patients with relapsed/refractory AML, and more interestingly, some patients achieved complete remissions (68). Other report revealed that CIML NK cell therapy induced complete remission in 47% of patients without inducing cytokine release syndrome, graft-versus-host disease, or immune cell-associated neurotoxicity syndrome (69). Also, it has been described that chemotherapy, followed by a donor lymphocyte infusion and the adoptive transfer of CIML NK cells induced clinical responses in pediatric AML patients that relapsed after hematopoietic cell transplantation (HCT) (70). Very recently, in AML patients receiving haploidentical HCT, infused CIML NK cells derived from the same donor persisted for a long time and were highly functional. More importantly, 87% of patients achieved a composite complete response (71). Another recent report described the therapeutic effect of CIML NK cell infusion to patients with relapsed myeloid diseases after haploidentical HCT. This study described that four of six patients showed clinical responses, and that three of them achieved a complete response (72). Therefore, current knowledge suggests that IL-12/15/18-preactivated NK cells may become a promising tool in cancer immunotherapy.

### What’s Next?

The large success and progress of chimeric antigen receptor (CAR)-engineered T cells in cancer immunotherapy (73) boosted the development of CAR-NK cells, which are currently being tested in multiple clinical trials (14, 14, 74). Interestingly, CAR technology can be combined with CIML NK cells to further enhance their functionality. CAR-engineered CIML NK cells showed enhanced antitumor responses against primary lymphoma targets *in vitro*. Moreover, these cells were able to significantly reduce tumor burden in a mouse lymphoma model and to prolong their survival (75). Of note, adoptive transfer of IL-12/15/18-preactivated NK cells is usually followed by the administration of IL-15 or IL-2 to support their survival and expansion, although cytokine support requires further characterization. Recent findings suggested that the infusion of IL-15 superagonist N-803 following adoptive CIML NK cell transfer may limit their clinical activity in comparison with IL-2 infusions (76). Remarkably, CAR-NK cells can be further modified to express and secrete cytokines such as IL-15 (77–82), so it would be interesting to explore if a therapy based on CAR-engineered CIML NK cells expressing IL-15 or IL-2 could further improve their efficacy.

A different approach that could enhance therapeutic efficiency of CIML NK cells is combining these cells with killer cell engagers and/or checkpoint inhibitors. As previously discussed, CIML NK cells transiently downregulate CD16 expression, but they are able to mediate ADCC. A recent study described that CIML NK cells from adults as well as IL-12/15/18-preactivated and expanded cord-blood derived NK cells can be loaded with the anti-CD30/CD16a bispecific antibody AFM13 and show antitumor activity *in vitro* and *in vivo* (83) (see also NCT04074746). Besides creating a thought-provoking debate about the most suitable NK cell source, this study suggested that CIML NK cells could be successfully combined with killer cell engagers, which represent a promising tool to boost NK cell functions (10). On the other hand, data reported by Berrien-Elliott et al. showed that NKG2A was upregulated in CIML NK cells and is associated with treatment failure in AML patients (69). Other studies also indicated that PD-1 expression can be induced in NK cells upon IL-12/15/18 stimulation in combination with glucocorticoids (84), or following a long (96 hours) IL-12/15/18 stimulation (85). Thus, it is tempting to speculate that CIML NK cell-based therapies could be also effectively combined with immune checkpoint inhibitors.

## Concluding Remarks

Memory-like responses of NK cells are a fascinating phenomenon that we are still trying to fully understand. Among them, CIML NK cells have drawn attention due to their superior antitumor properties and their therapeutic potential. Yet, many aspects of these cells remain unknown. Despite showing similarities with the trained immunity described in other innate immune cells, metabolic and epigenetic changes have been poorly characterized in IL-12/15/18-preactivated NK cells. Understanding their biology becomes even more challenging considering that memory-like behavior can be induced in NK cells from different sources (i.e. peripheral blood and umbilical cord blood). Interestingly, a recent report revealed that extracellular vesicles derived from IL-12/15/18-stimulated NK-92 cells showed higher ability to induce apoptosis in HCT116 colon cancer cell spheroids in comparison with IL-15-stimulated controls (86). These results suggests that NK-92 cells are somehow sensitive to IL-12/15/18 stimulation and thus it would be interesting to explore if memory-like behavior could be imprinted in NK cell lines. Independently of the source of CIML NK cells, the most relevant question is how could be further improved their therapeutic efficacy. Hopefully, new strategies such as genetically modifying CIML NK cells or combining them with other immunotherapeutic approaches could show durable clinical benefits. In any case, there is accumulating evidence that CIML NK cells are a valuable tool in cancer immunotherapy, and future research will reveal how to unlock their full potential.

## Author Contributions

All authors have made a substantial, direct and intellectual contribution to the work, and approved it for publication.

## Funding

Supported by the following grants: Fundación AECC-Spanish Association Against Cancer Foundation (PROYE16074BORR) and Health Department, Basque Government (2021333006). IT is recipient of a predoctoral contract funded by the Department of Education, Basque Government (PRE_2021_2_0215). GA-P and AA-I are recipient of grants from Jesús de Gangoiti Barrera Foundation (FJGB21/001 and FJBG21/005). GA-P is recipient of a predoctoral contract funded by Fundación AECC-Spanish Association Against Cancer Foundation (PRDVZ21440ASTA). FB is an Ikerbasque Research Professor, Ikerbasque, Basque Foundation for Science.

## Conflict of Interest

The authors declare that the research was conducted in the absence of any commercial or financial relationships that could be construed as a potential conflict of interest.

## Publisher’s Note

All claims expressed in this article are solely those of the authors and do not necessarily represent those of their affiliated organizations, or those of the publisher, the editors and the reviewers. Any product that may be evaluated in this article, or claim that may be made by its manufacturer, is not guaranteed or endorsed by the publisher.

## References

[1] Vivier E Artis D Colonna M Diefenbach A Di Santo JP Eberl G. Innate Lymphoid Cells: 10 Years on. Cell (2018) 174:1054–66. doi: 10.1016/j.cell.2018.07.017 30142344

[2] Chiossone L Dumas P-Y Vienne M Vivier E. Natural Killer Cells and Other Innate Lymphoid Cells in Cancer. Nat Rev Immunol (2018) 18:671–88. doi: 10.1038/s41577-018-0061-z 30209347

[3] Crinier A Kerdiles Y Vienne M Cózar B Vivier E Berruyer C. Multidimensional Molecular Controls Defining NK/ILC1 Identity in Cancers. Semin Immunol (2021) 52:101424. doi: 10.1016/j.smim.2020.101424 33272899

[4] Veluchamy JP Kok N Van der Vliet HJ Verheul HMW de Gruijl TD Spanholtz J. The Rise of Allogeneic Natural Killer Cells As a Platform for Cancer Immunotherapy: Recent Innovations and Future Developments. Front Immunol (2017) 8:631. doi: 10.3389/fimmu.2017.00631 28620386 PMC5450018

[5] Sanchez CE Dowlati EP Geiger AE Chaudhry K Tovar MA Bollard CM. NK Cell Adoptive Immunotherapy of Cancer: Evaluating Recognition Strategies and Overcoming Limitations. Transplant Cell Ther (2021) 27:21–35. doi: 10.1016/j.bbmt.2020.09.030 33007496 PMC8043250

[6] Terrén I Orrantia A Mikelez-Alonso I Vitallé J Zenarruzabeitia O Borrego F. NK Cell-Based Immunotherapy in Renal Cell Carcinoma. Cancers (Basel) (2020) 12:316. doi: 10.3390/cancers12020316 PMC7072691 32013092

[7] Khan M Arooj S Wang H. NK Cell-Based Immune Checkpoint Inhibition. Front Immunol (2020) 11:167. doi: 10.3389/fimmu.2020.00167 32117298 PMC7031489

[8] Mikelez-Alonso I Magadán S González-Fernández Á Borrego F. Natural Killer (NK) Cell-Based Immunotherapies and the Many Faces of NK Cell Memory: A Look Into How Nanoparticles Enhance NK Cell Activity. Adv Drug Delivery Rev (2021) 176:113860. doi: 10.1016/j.addr.2021.113860 34237404

[9] Myers JA Miller JS. Exploring the NK Cell Platform for Cancer Immunotherapy. Nat Rev Clin Oncol (2021) 18:85–100. doi: 10.1038/s41571-020-0426-7 32934330 PMC8316981

[10] Demaria O Gauthier L Debroas G Vivier E. Natural Killer Cell Engagers in Cancer Immunotherapy: Next Generation of Immuno-Oncology Treatments. Eur J Immunol (2021) 51:1934–42. doi: 10.1002/eji.202048953 34145579

[11] van Hall T André P Horowitz A Ruan DF Borst L Zerbib R. Monalizumab: Inhibiting the Novel Immune Checkpoint NKG2A. J Immunother Cancer (2019) 7:263. doi: 10.1186/s40425-019-0761-3 31623687 PMC6798508

[12] Molgora M Cortez VS Colonna M. Killing the Invaders: NK Cell Impact in Tumors and Anti-Tumor Therapy. Cancers (Basel) (2021) 13:595. doi: 10.3390/cancers13040595 33546248 PMC7913353

[13] Kang S Gao X Zhang L Yang E Li Y Yu L. The Advances and Challenges of NK Cell-Based Cancer Immunotherapy. Curr Oncol (2021) 28:1077–93. doi: 10.3390/curroncol28020105 PMC8025748 33652996

[14] Heipertz EL Zynda ER Stav-Noraas TE Hungler AD Boucher SE Kaur N. Current Perspectives on “Off-The-Shelf” Allogeneic NK and CAR-NK Cell Therapies. Front Immunol (2021) 12:732135. doi: 10.3389/fimmu.2021.732135 34925314 PMC8671166

[15] Becker PSA Suck G Nowakowska P Ullrich E Seifried E Bader P. Selection and Expansion of Natural Killer Cells for NK Cell-Based Immunotherapy. Cancer Immunol Immunother (2016) 65:477–84. doi: 10.1007/s00262-016-1792-y PMC4826432 26810567

[16] Mujal AM Delconte RB Sun JC. Natural Killer Cells: From Innate to Adaptive Features. Annu Rev Immunol (2021) 39:417–47. doi: 10.1146/annurev-immunol-101819-074948 33902312

[17] O’Leary JG Goodarzi M Drayton DL Von Andrian UH. T Cell– and B Cell–Independent Adaptive Immunity Mediated by Natural Killer Cells. Nat Immunol (2006) 7:507–16. doi: 10.1038/ni1332 16617337

[18] Stary V Stary G. NK Cell-Mediated Recall Responses: Memory-Like, Adaptive, or Antigen-Specific? Front Cell Infect Microbiol (2020) 10:208. doi: 10.3389/fcimb.2020.00208 32477964 PMC7240046

[19] Cooper MA Elliott JM Keyel PA Yang L Carrero JA Yokoyama WM. Cytokine-Induced Memory-Like Natural Killer Cells. Proc Natl Acad Sci USA (2009) 106:1915–9. doi: 10.1073/pnas.0813192106 PMC2644138 19181844

[20] Freud AG Mundy-Bosse BL Yu J Caligiuri MA. The Broad Spectrum of Human Natural Killer Cell Diversity. Immunity (2017) 47:820–33. doi: 10.1016/j.immuni.2017.10.008 PMC5728700 29166586

[21] Divangahi M Aaby P Khader SA Barreiro LB Bekkering S Chavakis T. Trained Immunity, Tolerance, Priming and Differentiation: Distinct Immunological Processes. Nat Immunol (2021) 22:2–6. doi: 10.1038/s41590-020-00845-6 33293712 PMC8020292

[22] Peng H Jiang X Chen Y Sojka DK Wei H Gao X. Liver-Resident NK Cells Confer Adaptive Immunity in Skin-Contact Inflammation. J Clin Invest (2013) 123:1444–56. doi: 10.1172/JCI66381 PMC3613925 23524967

[23] Wang X Peng H Cong J Wang X Lian Z Wei H. Memory Formation and Long-Term Maintenance of IL-7rα+ ILC1s *via* a Lymph Node-Liver Axis. Nat Commun (2018) 9:4854. doi: 10.1038/s41467-018-07405-5 30451860 PMC6242895

[24] Sun JC Beilke JN Lanier LL. Adaptive Immune Features of Natural Killer Cells. Nature (2009) 457:557–61. doi: 10.1038/nature07665 PMC2674434 19136945

[25] Hammer Q Rückert T Borst EM Dunst J Haubner A Durek P. Peptide-Specific Recognition of Human Cytomegalovirus Strains Controls Adaptive Natural Killer Cells. Nat Immunol (2018) 19:453–63. doi: 10.1038/s41590-018-0082-6 29632329

[26] Pahl JHW Koch J Götz J-J Arnold A Reusch U Gantke T. CD16A Activation of NK Cells Promotes NK Cell Proliferation and Memory-Like Cytotoxicity Against Cancer Cells. Cancer Immunol Res (2018) 6:517–27. doi: 10.1158/2326-6066.CIR-17-0550 29514797

[27] Rasid O Ciulean IS Fitting C Doyen N Cavaillon J-M. Local Microenvironment Controls the Compartmentalization of NK Cell Responses During Systemic Inflammation in Mice. J Immunol (2016) 197:2444–54. doi: 10.4049/jimmunol.1601040 27521338

[28] Rasid O Chevalier C Camarasa TM-N Fitting C Cavaillon J-M Hamon MA. H3K4me1 Supports Memory-Like NK Cells Induced by Systemic Inflammation. Cell Rep (2019) 29:3933–45.e3. doi: 10.1016/j.celrep.2019.11.043 31851924

[29] Lucas M Schachterle W Oberle K Aichele P Diefenbach A. Dendritic Cells Prime Natural Killer Cells by Trans-Presenting Interleukin 15. Immunity (2007) 26:503–17. doi: 10.1016/j.immuni.2007.03.006 PMC2084390 17398124

[30] Zanoni I Spreafico R Bodio C Di Gioia M Cigni C Broggi A. IL-15 Cis Presentation Is Required for Optimal NK Cell Activation in Lipopolysaccharide-Mediated Inflammatory Conditions. Cell Rep (2013) 4:1235–49. doi: 10.1016/j.celrep.2013.08.021 24055061

[31] Pal M Schwab L Yermakova A Mace EM Claus R Krahl AC. Tumor-Priming Converts NK Cells to Memory-Like NK Cells. Oncoimmunology (2017) 6:1–13. doi: 10.1080/2162402X.2017.1317411 PMC5486172 28680749

[32] North J Bakhsh I Marden C Pittman H Addison E Navarrete C. Tumor-Primed Human Natural Killer Cells Lyse NK-Resistant Tumor Targets: Evidence of a Two-Stage Process in Resting NK Cell Activation. J Immunol (2007) 178:85–94. doi: 10.4049/jimmunol.178.1.85 17182543

[33] Sabry M Tsirogianni M Bakhsh IA North J Sivakumaran J Giannopoulos K. Leukemic Priming of Resting NK Cells Is Killer Ig-Like Receptor Independent But Requires CD15-Mediated CD2 Ligation and Natural Cytotoxicity Receptors. J Immunol (2011) 187:6227–34. doi: 10.4049/jimmunol.1101640 22084431

[34] Sabry M Zubiak A Hood SP Simmonds P Arellano-Ballestero H Cournoyer E. Tumor- And Cytokine-Primed Human Natural Killer Cells Exhibit Distinct Phenotypic and Transcriptional Signatures. PloS One (2018) 14:1–20. doi: 10.1371/journal.pone.0218674 PMC6594622 31242243

[35] Granzin M Wagner J Köhl U Cerwenka A Huppert V Ullrich E. Shaping of Natural Killer Cell Antitumor Activity by Ex Vivo Cultivation. Front Immunol (2017) 8:458. doi: 10.3389/fimmu.2017.00458 28491060 PMC5405078

[36] Romee R Schneider SE Leong JW Chase JM Keppel CR Sullivan RP. Cytokine Activation Induces Human Memory-Like NK Cells. Blood (2012) 120:4751–60. doi: 10.1182/blood-2012-04-419283 PMC3520618 22983442

[37] Vendrame E Fukuyama J Strauss-Albee DM Holmes S Blish CA. Mass Cytometry Analytical Approaches Reveal Cytokine-Induced Changes in Natural Killer Cells. Cytom Part B - Clin Cytom (2017) 92:57–67. doi: 10.1002/cyto.b.21500 PMC5266600 27933717

[38] Ghofrani J Lucar O Dugan H Reeves RK Jost S. Semaphorin 7A Modulates Cytokine-Induced Memory-Like Responses by Human Natural Killer Cells. Eur J Immunol (2019) 49:1153–66. doi: 10.1002/eji.201847931 PMC6679804 31016720

[39] Romee R Foley B Lenvik T Wang Y Zhang B Ankarlo D. NK Cell CD16 Surface Expression and Function Is Regulated by a Disintegrin and Metalloprotease-17 (ADAM17). Blood (2013) 121:3599–608. doi: 10.1182/blood-2012-04-425397 PMC3643761 23487023

[40] Simhadri VR Dimitrova M Mariano JL Zenarruzabeitia O Zhong W Ozawa T. A Human Anti-M2 Antibody Mediates Antibody-Dependent Cell-Mediated Cytotoxicity (ADCC) and Cytokine Secretion by Resting and Cytokine-Preactivated Natural Killer (NK) Cells. PloS One (2015) 10:1–13. doi: 10.1371/journal.pone.0124677 PMC4411161 25915748

[41] Ewen E-M Pahl JHW Miller M Watzl C Cerwenka A. KIR Downregulation by IL-12/15/18 Unleashes Human NK Cells From KIR/HLA-I Inhibition and Enhances Killing of Tumor Cells. Eur J Immunol (2018) 48:355–65. doi: 10.1002/eji.201747128 29105756

[42] Lusty E Poznanski SM Kwofie K Mandur TS Lee DA Ashkar AA. IL-18/IL-15/IL-12 Synergy Induces Elevated and Prolonged IFN-γ Production by Ex Vivo Expanded NK Cells Which Is Not Due to Enhanced STAT4 Activation. Mol Immunol (2017) 88:138–47. doi: 10.1016/j.molimm.2017.06.025 28644973

[43] Wagner JA Berrien-Elliott MM Rosario M Leong JW Jewell BA Schappe T. Cytokine-Induced Memory-Like Differentiation Enhances Unlicensed Natural Killer Cell Antileukemia and Fcγriiia-Triggered Responses. Biol Blood Marrow Transplant (2017) 23:398–404. doi: 10.1016/j.bbmt.2016.11.018 27894857 PMC5408734

[44] Leong JW Chase JM Romee R Schneider SE Sullivan RP Cooper MA. Preactivation With IL-12, IL-15, and IL-18 Induces CD25 and a Functional High-Affinity IL-2 Receptor on Human Cytokine-Induced Memory-Like Natural Killer Cells. Biol Blood Marrow Transplant (2014) 20:463–73. doi: 10.1016/j.bbmt.2014.01.006 PMC3959288 24434782

[45] Terrén I Mikelez I Odriozola I Gredilla A González J Orrantia A. Implication of Interleukin-12/15/18 and Ruxolitinib in the Phenotype, Proliferation, and Polyfunctionality of Human Cytokine-Preactivated Natural Killer Cells. Front Immunol (2018) 9:737. doi: 10.3389/fimmu.2018.00737 29713323 PMC5911648

[46] Ni J Miller M Stojanovic A Garbi N Cerwenka A. Sustained Effector Function of IL-12/15/18–Preactivated NK Cells Against Established Tumors. J Exp Med (2012) 209:2351–65. doi: 10.1084/jem.20120944 PMC3526364 23209317

[47] Hüber CM Doisne J Colucci F. IL-12/15/18-Preactivated NK Cells Suppress GvHD in a Mouse Model of Mismatched Hematopoietic Cell Transplantation. Eur J Immunol (2015) 45:1727–35. doi: 10.1002/eji.201445200 PMC4687420 25778912

[48] Boieri M Ulvmoen A Sudworth A Lendrem C Collin M Dickinson AM. IL-12, IL-15, and IL-18 Pre-Activated NK Cells Target Resistant T Cell Acute Lymphoblastic Leukemia and Delay Leukemia Development *In Vivo* . Oncoimmunology (2017) 6:1–12. doi: 10.1080/2162402X.2016.1274478 PMC5384344 28405496

[49] Song Y Hu B Liu Y Jin Z Zhang Y Lin D. IL-12/IL-18-Preactivated Donor NK Cells Enhance GVL Effects and Mitigate GvHD After Allogeneic Hematopoietic Stem Cell Transplantation. Eur J Immunol (2018) 48:670–82. doi: 10.1002/eji.201747177 29282719

[50] Uppendahl LD Felices M Bendzick L Ryan C Kodal B Hinderlie P. Cytokine-Induced Memory-Like Natural Killer Cells Have Enhanced Function, Proliferation, and *In Vivo* Expansion Against Ovarian Cancer Cells. Gynecol Oncol (2019) 153:149–57. doi: 10.1016/j.ygyno.2019.01.006 PMC6430659 30658847

[51] Terrén I Orrantia A Vitallé J Astarloa-Pando G Zenarruzabeitia O Borrego F. Modulating NK Cell Metabolism for Cancer Immunotherapy. Semin Hematol (2020) 57:213–24. doi: 10.1053/j.seminhematol.2020.10.003 33256914

[52] Terrén I Orrantia A Mosteiro A Vitallé J Zenarruzabeitia O Borrego F. Metabolic Changes of Interleukin-12/15/18-Stimulated Human NK Cells. Sci Rep (2021) 11:6472. doi: 10.1038/s41598-021-85960-6 33742092 PMC7979769

[53] Surace L Doisne J-M Escoll P Marie S Dardalhon V Croft C. Polarized Mitochondria as Guardians of NK Cell Fitness. Blood Adv (2021) 5:26–38. doi: 10.1182/bloodadvances.2020003458 33570622 PMC7805327

[54] Becker-Hapak MK Shrestha N McClain E Dee MJ Chaturvedi P Leclerc GM. A Fusion Protein Complex That Combines IL-12, IL-15, and IL-18 Signaling to Induce Memory-Like NK Cells for Cancer Immunotherapy. Cancer Immunol Res (2021) 9:1071–87. doi: 10.1158/2326-6066.CIR-20-1002 PMC8416787 34244297

[55] Kedia-Mehta N Tobin L Zaiatz-Bittencourt V Pisarska MM De Barra C Choi C. Cytokine-Induced Natural Killer Cell Training Is Dependent on Cellular Metabolism and Is Defective in Obesity. Blood Adv (2021) 5:4447–55. doi: 10.1182/bloodadvances.2021005047 PMC8579258 34607345

[56] Assmann N O’Brien KL Donnelly RP Dyck L Zaiatz-Bittencourt V Loftus RM. Srebp-Controlled Glucose Metabolism Is Essential for NK Cell Functional Responses. Nat Immunol (2017) 18:1197–206. doi: 10.1038/ni.3838 28920951

[57] Sherwood ER Burelbach KR McBride MA Stothers CL Owen AM Hernandez A. Innate Immune Memory and the Host Response to Infection. J Immunol (2022) 208:785–92. doi: 10.4049/jimmunol.2101058 PMC8982914 35115374

[58] Lau CM Wiedemann GM Sun JC. Epigenetic Regulation of Natural Killer Cell Memory*. Immunol Rev (2022) 305:90–110. doi: 10.1111/imr.13031 34908173 PMC8955591

[59] Wiedemann GM Santosa EK Grassmann S Sheppard S Le Luduec J-B Adams NM. Deconvoluting Global Cytokine Signaling Networks in Natural Killer Cells. Nat Immunol (2021) 22:627–38. doi: 10.1038/s41590-021-00909-1 PMC8476180 33859404

[60] Ni J Hölsken O Miller M Hammer Q Luetke-Eversloh M Romagnani C. Adoptively Transferred Natural Killer Cells Maintain Long-Term Antitumor Activity by Epigenetic Imprinting and CD4 + T Cell Help. Oncoimmunology (2016) 5:e1219009. doi: 10.1080/2162402X.2016.1219009 27757318 PMC5048776

[61] Zhang C Yin J Zheng J Xiao J Hu J Su Y. EZH2 Identifies the Precursors of Human Natural Killer Cells With Trained Immunity. Cancer Biol Med (2021) 18:1021–39. doi: 10.20892/j.issn.2095-3941.2020.0791 PMC8610163 34553850

[62] Keppel MP Yang L Cooper MA. Murine NK Cell Intrinsic Cytokine-Induced Memory-Like Responses Are Maintained Following Homeostatic Proliferation. J Immunol (2013) 190:4754–62. doi: 10.4049/jimmunol.1201742 PMC3633638 23530145

[63] Simhadri VR Mariano JL Zenarruzabeitia O Seroogy CM Holland SM Kuehn HS. Intact IL-12 Signaling Is Necessary for the Generation of Human Natural Killer Cells With Enhanced Effector Function After Restimulation. J Allergy Clin Immunol (2014) 134:1190–3.e1. doi: 10.1016/j.jaci.2014.06.006 PMC4252978 25065718

[64] Cubitt CC McClain E Becker-Hapak M Foltz JA Wong P Wagner JA. A Novel Fusion Protein Scaffold 18/12/TxM Activates the IL-12, IL-15, and IL-18 Receptors to Induce Human Memory-Like Natural Killer Cells. Mol Ther - Oncolytics (2022) 24:585–96. doi: 10.1016/j.omto.2022.02.009 PMC8889352 35284622

[65] Zhuang L Fulton RJ Rettman P Sayan AE Coad J Al-Shamkhani A. Activity of IL-12/15/18 Primed Natural Killer Cells Against Hepatocellular Carcinoma. Hepatol Int (2019) 13:75–83. doi: 10.1007/s12072-018-9909-3 30467624 PMC6513806

[66] Bonanni V Antonangeli F Santoni A Bernardini G. Targeting of CXCR3 Improves Anti-Myeloma Efficacy of Adoptively Transferred Activated Natural Killer Cells. J Immunother Cancer (2019) 7:1–16. doi: 10.1186/s40425-019-0751-5 31699153 PMC6839099

[67] Marin ND Krasnick BA Becker-Hapak M Conant L Goedegebuure SP Berrien-Elliott MM. Memory-Like Differentiation Enhances NK Cell Responses to Melanoma. Clin Cancer Res (2021) 27:4859–69. doi: 10.1158/1078-0432.CCR-21-0851 PMC8416927 34187852

[68] Romee R Rosario M Berrien-Elliott MM Wagner JA Jewell BA Schappe T. Cytokine-Induced Memory-Like Natural Killer Cells Exhibit Enhanced Responses Against Myeloid Leukemia. Sci Transl Med (2016) 8:357ra123. doi: 10.1126/scitranslmed.aaf2341 PMC5436500 27655849

[69] Berrien-Elliott MM Cashen AF Cubitt CC Neal CC Wong P Wagner JA. Multidimensional Analyses of Donor Memory-Like NK Cells Reveal New Associations With Response After Adoptive Immunotherapy for Leukemia. Cancer Discov (2020) 10:1854–71. doi: 10.1158/2159-8290.CD-20-0312 PMC7710923 32826231

[70] Bednarski JJ Zimmerman C Berrien-Elliott MM Foltz JA Becker-Hapak M Neal CC. Donor Memory-Like NK Cells Persist and Induce Remissions in Pediatric Patients With Relapsed AML After Transplant. Blood (2022) 139:1670–83. doi: 10.1182/blood.2021013972 PMC8931511 34871371

[71] Berrien-Elliott MM Foltz JA Russler-Germain DA Neal CC Tran J Gang M. Hematopoietic Cell Transplantation Donor-Derived Memory-Like NK Cells Functionally Persist After Transfer Into Patients With Leukemia. Sci Transl Med (2022) 14:eabm1375. doi: 10.1126/scitranslmed.abm1375 35196021 PMC9210521

[72] Shapiro RM Birch GC Hu G Vergara Cadavid J Nikiforow S Baginska J. Expansion, Persistence, and Efficacy of Donor Memory-Like NK Cells Infused for Post-Transplant Relapse. J Clin Invest (2022) e154334. doi: 10.1172/JCI154334 35349491 PMC9151697

[73] De Bousser E Callewaert N Festjens N. T Cell Engaging Immunotherapies, Highlighting Chimeric Antigen Receptor (CAR) T Cell Therapy. Cancers (Basel) (2021) 13:6067. doi: 10.3390/cancers13236067 34885176 PMC8657024

[74] Khawar MB Sun H. CAR-NK Cells: From Natural Basis to Design for Kill. Front Immunol (2021) 12:707542. doi: 10.3389/fimmu.2021.707542 34970253 PMC8712563

[75] Gang M Marin ND Wong P Neal CC Marsala L Foster M. CAR-Modified Memory-Like NK Cells Exhibit Potent Responses to NK-Resistant Lymphomas. Blood (2020) 136:2308–18. doi: 10.1182/blood.2020006619 PMC7702478 32614951

[76] Berrien-Elliott MM Becker-Hapak M Cashen AF Jacobs MT Wong P Foster M. Systemic IL-15 Promotes Allogeneic Cell Rejection in Patients Treated With Natural Killer Cell Adoptive Therapy. Blood (2022) 139:1177–83. doi: 10.1182/blood.2021011532 PMC9211446 34797911

[77] Liu E Tong Y Dotti G Shaim H Savoldo B Mukherjee M. Cord Blood NK Cells Engineered to Express IL-15 and a CD19-Targeted CAR Show Long-Term Persistence and Potent Antitumor Activity. Leukemia (2018) 32:520–31. doi: 10.1038/leu.2017.226 PMC6063081 28725044

[78] Liu E Marin D Banerjee P Macapinlac HA Thompson P Basar R. Use of CAR-Transduced Natural Killer Cells in CD19-Positive Lymphoid Tumors. N Engl J Med (2020) 382:545–53. doi: 10.1056/NEJMoa1910607 PMC7101242 32023374

[79] Wang X Jasinski DL Medina JL Spencer DM Foster AE Bayle JH. Inducible MyD88/CD40 Synergizes With IL-15 to Enhance Antitumor Efficacy of CAR-NK Cells. Blood Adv (2020) 4:1950–64. doi: 10.1182/bloodadvances.2020001510 PMC7218419 32384544

[80] Daher M Basar R Gokdemir E Baran N Uprety N Nunez Cortes AK. Targeting a Cytokine Checkpoint Enhances the Fitness of Armored Cord Blood CAR-NK Cells. Blood (2021) 137:624–36. doi: 10.1182/blood.2020007748 PMC7869185 32902645

[81] Du Z Ng YY Zha S Wang S. Piggybac System to Co-Express NKG2D CAR and IL-15 to Augment the *In Vivo* Persistence and Anti-AML Activity of Human Peripheral Blood NK Cells. Mol Ther - Methods Clin Dev (2021) 23:582–96. doi: 10.1016/j.omtm.2021.10.014 PMC8609108 34853803

[82] Christodoulou I Ho WJ Marple A Ravich JW Tam A Rahnama R. Engineering CAR-NK Cells to Secrete IL-15 Sustains Their Anti-AML Functionality But Is Associated With Systemic Toxicities. J Immunother Cancer (2021) 9:e003894. doi: 10.1136/jitc-2021-003894 34896980 PMC8655609

[83] Kerbauy LN Marin ND Kaplan M Banerjee PP Berrien-Elliott MM Becker-Hapak M. Combining AFM13, a Bispecific CD30/CD16 Antibody, With Cytokine-Activated Blood and Cord Blood–Derived NK Cells Facilitates CAR-Like Responses Against CD30+ Malignancies. Clin Cancer Res (2021) 27:3744–56. doi: 10.1158/1078-0432.CCR-21-0164 PMC8254785 33986022

[84] Quatrini L Vacca P Tumino N Besi F Di Pace AL Scordamaglia F. Glucocorticoids and the Cytokines IL-12, IL-15, and IL-18 Present in the Tumor Microenvironment Induce PD-1 Expression on Human Natural Killer Cells. J Allergy Clin Immunol (2021) 147:349–60. doi: 10.1016/j.jaci.2020.04.044 32417134

[85] Wagner A Kadri N Tibbitt C Van de Ven K Bagawath-Singh S Oliinyk D. PD-1 Expression on NK Cells can be Related to Cytokine Stimulation and Tissue Residency. bioRxiv (2021). doi: 10.1101/2021.03.29.437486

[86] Aarsund M Segers FM Wu Y Inngjerdingen M. Comparison of Characteristics and Tumor Targeting Properties of Extracellular Vesicles Derived From Primary NK Cells or NK-Cell Lines Stimulated With IL-15 or IL-12/15/18. Cancer Immunol Immunother (2022). (online preprint) doi: 10.1007/s00262-022-03161-0 PMC9374793 35119498

